# A comparison of machine learning methods for predicting recurrence and death after curative-intent radiotherapy for non-small cell lung cancer: Development and validation of multivariable clinical prediction models

**DOI:** 10.1016/j.ebiom.2022.103911

**Published:** 2022-03-03

**Authors:** Sumeet Hindocha, Thomas G. Charlton, Kristofer Linton-Reid, Benjamin Hunter, Charleen Chan, Merina Ahmed, Emily J. Robinson, Matthew Orton, Shahreen Ahmad, Fiona McDonald, Imogen Locke, Danielle Power, Matthew Blackledge, Richard W. Lee, Eric O. Aboagye

**Affiliations:** a Lung Unit, The Royal Marsden NHS Foundation Trust, Fulham Road, London SW36JJ, UK; b AI for Healthcare Centre for Doctoral Training, Imperial College London, Exhibition Road, London SW7 2BX, UK; c Department of Clinical Oncology, Institute of Cancer Research NIHR Biomedical Research Centre, London, UK; d Cancer Imaging Centre, Department of Surgery and Cancer, Imperial College London, Du Cane Road, London W12 0NN, UK; e Guy's Cancer Centre, Guy's and St Thomas’ NHS Foundation Trust, Great Maze Pond, London SE19RT UK; f Lung Unit, The Royal Marsden NHS Foundation Trust, Downs Road, Sutton SM25PT, UK; g Clinical Trials Unit, Royal Marsden NHS Foundation Trust, Downs Road, Sutton SM25PT, UK; h Artificial Intelligence Imaging Hub, Royal Marsden NHS Foundation Trust, Downs Road, Sutton SM25PT, UK; i Department of Clinical Oncology, Charing Cross Hospital, Fulham Palace Road, London W6 8RF, UK; j Radiotherapy and Imaging, Institute of Cancer Research, 123 Old Brompton Road, London SW7 3RP, UK; k Early Diagnosis and Detection Centre, National Institute for Health Research (NIHR) Biomedical Research Centre at The Royal Marsden NHS Foundation Trust and the Institute of Cancer Research, London; l National Heart and Lung Institute, Imperial College, London, UK

**Keywords:** Non-small cell lung cancer, Radiotherapy, Machine learning, Recurrence, Overall survival, Prediction, Early detection

## Abstract

**Background:**

Surveillance is universally recommended for non-small cell lung cancer (NSCLC) patients treated with curative-intent radiotherapy. High-quality evidence to inform optimal surveillance strategies is lacking. Machine learning demonstrates promise in accurate outcome prediction for a variety of health conditions. The purpose of this study was to utilise readily available patient, tumour, and treatment data to develop, validate and externally test machine learning models for predicting recurrence, recurrence-free survival (RFS) and overall survival (OS) at 2 years from treatment.

**Methods:**

A retrospective, multicentre study of patients receiving curative-intent radiotherapy for NSCLC was undertaken. A total of 657 patients from 5 hospitals were eligible for inclusion. Data pre-processing derived 34 features for predictive modelling. Combinations of 8 feature reduction methods and 10 machine learning classification algorithms were compared, producing risk-stratification models for predicting recurrence, RFS and OS. Models were compared with 10-fold cross validation and an external test set and benchmarked against TNM-stage and performance status. Youden Index was derived from validation set ROC curves to distinguish high and low risk groups and Kaplan-Meier analyses performed.

**Findings:**

Median follow-up time was 852 days. Parameters were well matched across training-validation and external test sets: Mean age was 73 and 71 respectively, and recurrence, RFS and OS rates at 2 years were 43% vs 34%, 54% vs 47% and 54% vs 47% respectively. The respective validation and test set AUCs were as follows: 1) RFS: 0·682 (0·575–0·788) and 0·681 (0·597–0·766), 2) Recurrence: 0·687 (0·582–0·793) and 0·722 (0·635–0·81), and 3) OS: 0·759 (0·663–0·855) and 0·717 (0·634–0·8). Our models were superior to TNM stage and performance status in predicting recurrence and OS.

**Interpretation:**

This robust and ready to use machine learning method, validated and externally tested, sets the stage for future clinical trials entailing quantitative personalised risk-stratification and surveillance following curative-intent radiotherapy for NSCLC.

**Funding:**

A full list of funding bodies that contributed to this study can be found in the Acknowledgements section.

**Research in context**

**Evidence before this study**

We searched PubMed Central for papers published from database inception to 6^th^ June 2020, with the terms “non-small cell lung cancer”, “radiotherapy”, “machine learning”, “prognosis”, “outcome” and “prediction” with no language restrictions. This returned 809 results which were manually screened for suitability. The majority of returned results either did not involve NSCLC or pertained to imaging (radiomic and deep-learning) or biological models which was not our focus. Six studies related to our work.

To the best of our knowledge, no published studies to date have used routinely available clinical data to compare multiple machine learning methods to build prediction models for recurrence and survival following curative-intent radiotherapy in non-small cell lung cancer.

Previous studies using clinical data for outcome prediction following radiotherapy for NSCLC have explored cox proportional-hazards models and support vector machines and have generally focused on overall survival (OS), with few studies looking at recurrence or recurrence-free survival (RFS). This is likely due to accurate data on death being more readily accessible from national-level registries than recurrence data; however recurrence and RFS may offer more clinical utility than OS in stratifying follow-up, allowing clinicians to potentially intervene earlier to provide further, potentially curable treatment.

**Added value of this study**

Our study is the first to compare multiple machine learning algorithms and feature reduction methods using routinely available clinical data and to develop, validate and externally test prediction models for recurrence, RFS and OS following radical radiotherapy for NSCLC. Such approaches may overcome the limitations of traditional statistical methods such as Cox proportional hazards models.

**Implications of all the available evidence**

Performance of our models exceed traditional methods and show consistency across validation and external test sets. This robust and readily replicable machine learning method, validated and externally tested, sets the stage for future clinical trials entailing quantitative personalised risk-stratification and surveillance following curative-intent radiotherapy for NSCLC.

Alt-text: Unlabelled box

## Introduction

Lung cancer is the leading cause of cancer deaths worldwide.^1^ Non-small cell lung cancer (NSCLC) accounts for 85% of lung cancers, with approximately 1 in 5 patients alive 5 years after diagnosis.^2^ Recurrence is reported in up to 36% of patients receiving curative-intent treatment for NSCLC.^3^ Surveillance is recommended across international guidelines for NSCLC patients treated with curative-intent radiotherapy.^3^ Surveillance ensures on-going patient support, management of co-morbidities and cancer treatment-related side effects as well as detection of recurrence of the treated cancer or second (metachronous) primary cancers. Curative treatment following local recurrence results in 5-year survival rates of 15%.^4^ Earlier detection of recurrence may therefore improve survival and quality of life. Similarly, surveillance-stratification may allow for better resource allocation.

International guidance highlights a lack of high-quality evidence to formulate specific recommendations on the nature and frequency of follow-up after radiotherapy.^3^ Most guidance is based on expert consensus and advises follow-up with clinical review and contrast-enhanced chest CT at 3–6 monthly intervals for 2–3 years. Without an agreed framework, follow-up differs according to local policy or even individual clinician opinion.

The UK's National Institute of Healthcare and Clinical Excellence (NICE) have recommended further research into use of prognostic factors to develop risk-stratification models to inform optimal surveillance.^4^ Risk-stratification models may allow for personalised follow-up for NSCLC patients undergoing curative-intent radiotherapy, resulting in potentially earlier detection of recurrence for high-risk patients or avoidance of unnecessary scans and hospital visits for low-risk patients. Such models would improve patient care and healthcare resource management.

Previous studies using clinical data for outcome prediction following radiotherapy for NSCLC have explored cox proportional-hazards models and support vector machines, and have generally focused on OS, achieving validation or external test set AUCs between 0·61 and 0·69 and C-Index scores between 0·58 and 0·69.^3^^,^^5^, ^6^, ^7^, ^8^ It is possible that more advanced machine learning techniques can better handle large clinical datasets leading to superior performance. Comparison of the performance of several machine learning models has been undertaken for systemic anti-cancer therapy for advanced NSCLC,^9^ but is lacking in the radical radiotherapy cohort.

The intent of this study was therefore to utilise a broad range of readily available patient, tumour, and treatment related features in comparing performance of several machine learning methods to develop and validate prediction models for recurrence, recurrence-free survival (RFS) and overall survival (OS). Such models may be used to guide personalised risk-stratification and surveillance following curative-intent radiotherapy for NSCLC in future.

## Methods

### Ethics

This study was approved by the UK Health Research Authority (reference number: 20/HRA/3051), ClinicalTrials.gov identifier: NCT04721444.

### Datasets

We utilised 3 independent, novel datasets of patients receiving primary curative-intent radiotherapy for stage I to III NSCLC, yielding a total of 722 patients across 5 hospitals.

Each dataset was retrospectively collated from electronic patient record (EPR) and radiotherapy treatment planning systems (TPS) at UK National Health Service (NHS) Trusts:

- Dataset X consists of 434 patients with stage I to III disease treated at X Trust with stereotactic or conventional radiotherapy with or without chemotherapy between 26/9/2014 and 23/10/2018.
- Dataset Y consists of 111 patients with stage I to III disease treated at Y Trust with conventional radiotherapy with or without chemotherapy between 3/2/2014 and 10/1/2019.
- Dataset Z consists of 177 patients with stage I to III disease treated at Z Trust with stereotactic or conventional radiotherapy with or without chemotherapy between 21/1/2016 and 18/12/2018.

The datasets with the most (Dataset X) and least (Dataset Y) patients were combined and randomly divided into training and validation sets. The testing set (Dataset Z) was selected by ring-fencing one specific site as a geographically external test set. The data were collected in early 2021, ensuring a minimum of 2 years of follow-up for all patients. Those with no known recurrence or death within 60 days of the 2-year endpoint, or no recurrence within 60 days of death, were taken to have no event. This cut-off was agreed upon by the authors after discussion about how best to reduce bias and factoring in the variable nature of clinical follow-up appointments. 60 days was taken to be mean timeframe between follow-up appointments and thus an estimated half-way point between the last time a patient was seen and the 2-year endpoint. Therefore, we agreed that if a patient had no known recurrence or death within 60 days of the endpoint, or no recurrence within 60 days of death, it was unlikely that a recurrence/death had occurred.

The following patient demographics and clinical parameters were collected: sex, age, ethnicity, World Health Organisation (WHO) performance status, smoking status, TNM8 T-stage, TNM8 N-stage, TNM8 overall clinical stage, size of primary lesion, FDG PET-CT Standard Uptake Value (SUV) of primary lesion, nodal avidity and maximal nodal SUV, whether nodes were sampled (e.g. with endoscopic bronchial ultrasound, EBUS), whether there was a confirmed pathological diagnosis (e.g. with biopsy) and histological type, body mass index, pre-treatment forced expiratory volume in 1 second (FEV1, as percent predicted) and diffusing capacity for carbon monoxide (TLCO, as percent predicted), pre and post-treatment neutrophil and lymphocyte counts, type of radiotherapy treatment received (stereotactic body radiotherapy (SBRT) or conventional radiotherapy with or without chemotherapy), total dose in Gy, number of fractions, biologically effective dose in Gy (assuming an α/β value of 10) radiotherapy gross tumour volume (GTV), radiotherapy planning target volume (PTV) and dates of radiotherapy planning scan and first and last fraction of radiotherapy.

To meet pre-processing requirements for machine learning, categorical data were converted to numeric. One-hot-encoding converted each level of each categorical feature into a new binary feature. To mitigate for resultant increase in data dimensionality, prior to one-hot-encoding, levels of some categorical features were combined, for example, “never”, “ex” and “current” smokers were binarized to “never” and “ever”. Implicit associations between variables were made explicit: dates of planning scan and first and last fraction were replaced with the number of days between them. Missing clinical data were assumed missing at random and non-dependent on outcome. Features with more than 25% of observations missing were removed. Missing data for remaining features was imputed using the multiple imputation with chained equations (MICE) package with default arguments in R. Highly correlated features were removed using the treatment_corr function, with a threshold of 0·85, removing one of each pair of correlated features (Pearson correlation for continuous and Spearman correlation for categorical features). Continuous features were standardized.

### Statistics

Patient demographics and clinical parameters are summarised as means and standard deviations for continuous features, and frequencies and percentages for categorical features. Comparisons between datasets are summarised using Wilcoxon rank sum test for continuous features and Fisher exact test for categorical features. Time to event data for each of the study outcomes (recurrence, RFS and OS) were binarized at 2 years from the first fraction of radiotherapy for classification purposes – cases scored “1” if there was recurrence or death within 2 years, and “0” otherwise. Owing to the nature of clinical follow-up, 340 patients (47%) were not seen at or after the 2-year endpoint. As simply excluding these patients would bias the dataset, those with no known recurrence or death within 60 days of the endpoint, or no recurrence within 60 days of death, were taken to have no event. Those last seen more than 60 days from the endpoint or date of death were excluded (*n* = 65, 9%). More information can be found in the Supplementary Material section “Further details on datasets”. To ensure non-biased dataset assignment for training, validation, and testing, the datasets with the most (Dataset X) and least patients (Dataset Y) were combined and then cases randomly assigned as training and validation with an 80:20 ratio, stratified by the binarized outcome. The Z dataset was locked for testing.

### Feature handling and modelling

Dimensionality reduction may be required prior to modelling to increase prediction accuracy, prevent overfitting, and reduce computational cost. We explored a combination of 10 linear, Bayesian, neural-net and tree-based machine learning algorithms applied to 8 different feature sets (either no feature reduction or following 1 of 7 feature reduction methods, where possible. Feature reduction methods included correlation-based, multivariate linear penalised and recursive approaches). A brief description of these algorithms, feature reduction methods and resultant feature sets can be found in Supplementary Table 2. Hyper-parameter optimisation was performed via grid-search with 3 repeats of 10-fold cross-validation using the caret package in R. Hyper-parameters of the final selected models are listed in Supplementary Table 1.

Receiver-Operator Characteristic (ROC) curves were created for the validation set results of each algorithm-feature set combination and the Area Under the Curve (AUC) calculated. Ensemble prediction models were then explored by averaging the predictions of the 3 algorithms with the highest AUC in the validation set for each particular outcome being predicted. Where the ensemble model was superior, it was selected as the final model for predicting that particular outcome on the external test set. Otherwise, the single algorithm with the highest AUC was selected as the final model.

Caret's varImp function^10^ was used to identify features that contributed most significantly to model performance. It provides a generic method for calculating variable importance by either utilising a model's native feature importance ranking method or using ROC curve analysis for each feature.^10^ It is not possible to do this for averaged predictions across algorithms and so where an ensemble was selected as the final model, this was performed for the component algorithms instead.

### Benchmarking

TNM-stage and performance status are known prognostic factors in NSCLC.^11^^,^^12^ Logistic regression models based on TNM stage and performance status were developed to benchmark our prediction models against. As above, the Caret package was used with hyper-parameter optimisation performed via grid-search with 3 repeats of 10-fold cross-validation. AUC was calculated.

### Risk-groups

Youden's Index was calculated from the validation set ROC curve for each final model and used to create a classification threshold for outcome prediction. This threshold was also used to separate groups into high (outcome event occurs within 2 years of first fraction of radiotherapy) or low (outcome does not occur within 2 years of first fraction of radiotherapy) risk groups. Time to event data were then used to create Kaplan Meier curves demonstrating the difference in recurrence/RFS/OS between high and low risk groups. Log-rank test with a significance level of 0·05 was used to determine difference between survival curves. Performance of the risk models was assessed using the external test set. Using the same threshold as for Kaplan-Meier analysis, classification was performed for each outcome. Balanced accuracy and F1 score were recorded, in order to convey performance in the context of imbalanced endpoints. All analyses were carried out using R 3.5.1.

### Role of the funding source

The funding source played no role in study design, data collection, data analysis, data interpretation, or writing of the report. All authors had full access to all the data in the study and the corresponding authors had final responsibility for the decision to submit for publication.

## Results

In total, 657 patients were included in the study. Median follow-up was 852 days. The combined training-validation and external test sets included 498 and 159 patients respectively. Patient demographics and clinical parameters are a summarised in Table 1.

**Table 1:** Demographic and clinical parameters for combined training-validation and external test sets (prior to imputation). Features not used for modelling are not shown. Categorical data are summarised with means and percentages and *p*-values pertain to Fishers exact test. Continuous data are summarised with median and inter-quartile range (IQR) and *p*-values pertain to Wilcoxon rank sum test.

Parameter	Combined Training & Validation Sets *n* = 498	External Test Set *n* = 159	*P*-value
Age (IQR)	74 (14)	72 (14)	·054
Sex (%) - Male - Female	273 (54·8) 225 (45·2)	88 (55·4) 71 (44·7)	·907
WHO Performance Status (%) - 0 - 1 - 2 - Missing	82 (16·5) 282 (56·6) 118 (23·7) 16 (3·2)	16 (10·1) 85 (53·5) 55 (34·6) 3 (1·9)	·023
Body Mass Index (IQR) - Missing, n (%)	25·1 (6.5) 103 (20·7)	26·22 (7·1) 7 (4·4)	·044
Smoking Status (%) - Never - Ever - Missing	42 (8·4) 437 (87·8) 19 (3·8)	8 (5·0) 141 (88·7) 10 (6·3)	·165
TNM8 T stage (%) - 1 - 2 - 3 - 4	192 (38·6) 133 (26·7) 74 (14·9) 99 (19·9)	77 (48·4) 36 (22·6) 17 (10·7) 29 (18·2)	·161
TNM8 N stage (%) - 0 - 1 - 2 - 3	276 (55·4) 50 (10·0) 130 (26·1) 42 (8·4)	106 (66·8) 7 (4·4) 35 (22·0) 11 (6·9)	·041
FEV1, percent predicted (IQR) - Missing, n (%)	76 (33.2) 46 (9·2)	68·5 (34·5) 17 (10·7)	·004
TLCO, percent predicted (IQR) - Missing, n (%)	60 (25) 79 (15·9)	57 (25·8) 25 (15·7)	·092
Days from planning scan to first fraction (IQR)	18 (7)	18 (6·0)	·856
Size of primary (IQR) - Missing, n (%)	33 (28) 16 (3·2)	30 (28·5) 0	·050
SUV primary (IQR) - Missing, n (%)	10·35 (8·9) 54 (10·8)	9·3 (9·8) 8 (5·0)	·829
Max nodal SUV (IQR) - Missing, n (%)	7·35 (6·8) 63 (12·7)	5·7 (4·7) 48 (30·2)	<·001
Nodal avidity (%) - Yes - No - Missing	194 (39·0) 286 (57·4) 18 (3·6)	67 (42·1) 92 (57·9) 0	·024
Nodal Sampling (%) - Yes - No - Missing	159 (31·9) 334 (67·1) 5 (1·0)	57 (35·9) 99 (62·3) 3 (1·9)	·380
Histology (%) - Adenocarcinoma - Squamous - Other - No pathology	223 (44·8) 158 (31·7) 49 (9·8) 68 (13·7)	59 (37·1) 56 (35·2) 5 (3·1) 39 (24·5)	<·001
Treatment type (%) - SBRT - Conventional RT - Chemo + RT	174 (34·9) 125 (25·1) 199 (40·0)	73 (45·9) 33 (20·8) 53 (33·3)	·045
Number of fractions (IQR)	20 (27)	20 (27)	·402
Total Dose, Gy (IQR)	55 (9)	55 (9)	·137
Biologically Effective Dose, Gy (IQR)	76·8 (45·4)	76·8 (38·7)	·023
Planning Target Volume, cm3 (IQR)	218·39 (343·2)	126·62 (314·3)	·097
Recurrence at 2 years (%)	214 (43·0)	54 (34·0)	·051
Recurrence or death at 2 years (%)	267 (53·6)	74 (46·5)	·122
Death at 2 years (%)	185 (37·2)	54 (34·0)	·508
Median length of follow-up (range)	836 (0–2462)	868 (0–1442)	

Datasets were well-matched for most parameters. The external test set had a higher proportion of patients with performance status 2 (34·6% vs 23·7%) and with earlier stage disease (TNM8 T1-stage 1 48·4% vs 38·6%, N0-stage 66·7% vs 55·4%). There were fewer cases of adenocarcinoma (37·1% vs 44·8%) and more patients lacking histological diagnosis (24·5% vs 13·7%) and receiving SBRT (45·9% vs 34·9%) in the external test set. The recurrence, RFS and OS endpoints were marginally imbalanced in the validation and external test sets.^13^

**Figure 1:**
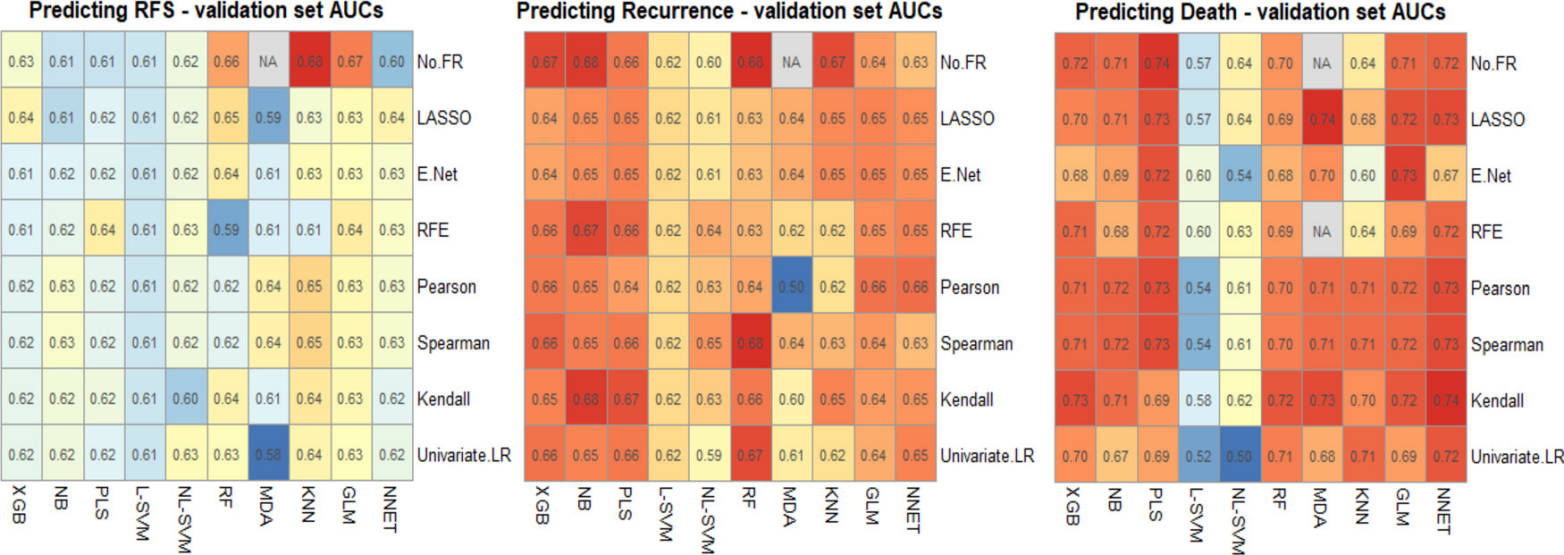
Heatmaps illustrating the performance of each machine learning algorithm (rows) with each feature reduction method (columns), measured by validation set AUC. No FR: No feature reduction (full feature set used), LASSO: Least Absolute Shrinkage and Selection Operator, E Net: Elastic-Net, RFE: Recursive Feature Elimination, Univariate LR: Univariate Logistic Regression, XGB: Extreme Gradient Boosting machine, NB: Naïve-Bayes, PSL: Partial Least Squares, L-SVM: Linear Support Vector Machine, NL-SVM: Non-linear (radial) SVM, RF: Random Forest, MDA: Mixture Discriminant Analysis, KNN: K-Nearest Neighbours, GLM: Generalised Linear Model, NNET: Neural Network.

Following data pre-processing a total of 12 continuous and 22 discrete features were used for machine learning. These are listed in Supplementary Table 2, together with the features that remained after each feature reduction method for each of the measured outcomes. The results of our experiments with machine learning algorithms and feature set combinations on the validation set for each outcome are shown in Figure 1. These are in the form of heatmaps showing the AUC for each combination of machine learning algorithm (rows) with feature selection method (columns).

Averaging across the machine learning algorithms (columns), the best performing feature sets for predicting RFS and recurrence were the full sets with no further feature reduction and for predicting death, the best performing feature set was after Kendall's rank correlation.

**Figure 2:**
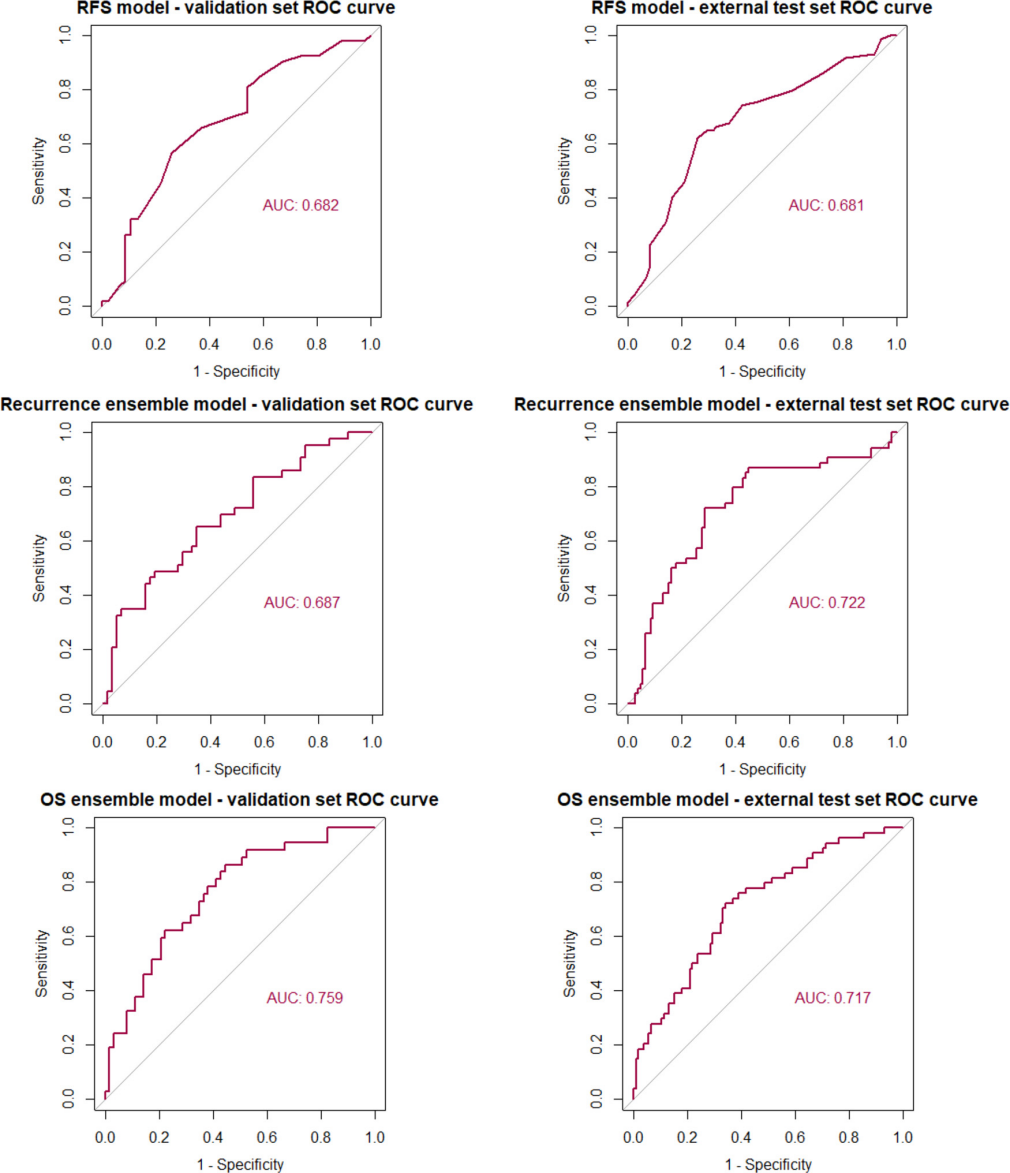
ROC curves for the validation and external test set for each prediction.

The final prediction models chosen were as follows: For RFS, KNN alone, for recurrence, an ensemble of NB, RF and KNN, and for OS, an ensemble of MDA, XGB and NNET. Results of the final models on validation and external test sets are shown in Figure 2 (ROC curves) and Table 2 (AUCs with 95% confidence intervals), together with a comparison against both the TNM stage and the performance status-based benchmarking models. Performance of our models was consistent across both validation and external test sets and whilst there was some overlap in confidence intervals with the benchmarking models, absolute AUC was superior for predicting recurrence and OS. For predicting RFS, our model was superior to the performance status-based model in both validation and external test sets, however, was eclipsed by the TNM stage-based model in external test set performance.

**Table 2:** AUC with 95% confidence intervals for the validation and external test set for each prediction model, benchmarked against models based on TNM-stage and performance status.

Outcome		Validation Set		External Test Set	
		AUC	95% CI	AUC	95% CI
RFS	**Our** prediction model	0·682	0·575–0·788	0·681	0·597–0·766
	**TNM based model**	0·650	0·541–0·760	0·695	0·616–0·774
	**PS based model**	0·464	0·363–0·565	0·499	0·418–0·58
Recurrence	**Our** prediction model	0·687	0·582–0·793	0·722	0·635–0·810
	**TNM based model**	0·670	0·563–0·777	0·707	0·622–0·791
	**PS based model**	0·506	0·402–0·609	0·584	0·503–0·665
OS	**Our** prediction model	0·759	0·663–0·855	0·717	0·634–0·800
	**TNM based model**	0·649	0·541–0·756	0·665	0·579–0·751
	**PS based model**	0·459	0·357–0·561	0·531	0·447–0·615

**Figure 3:**
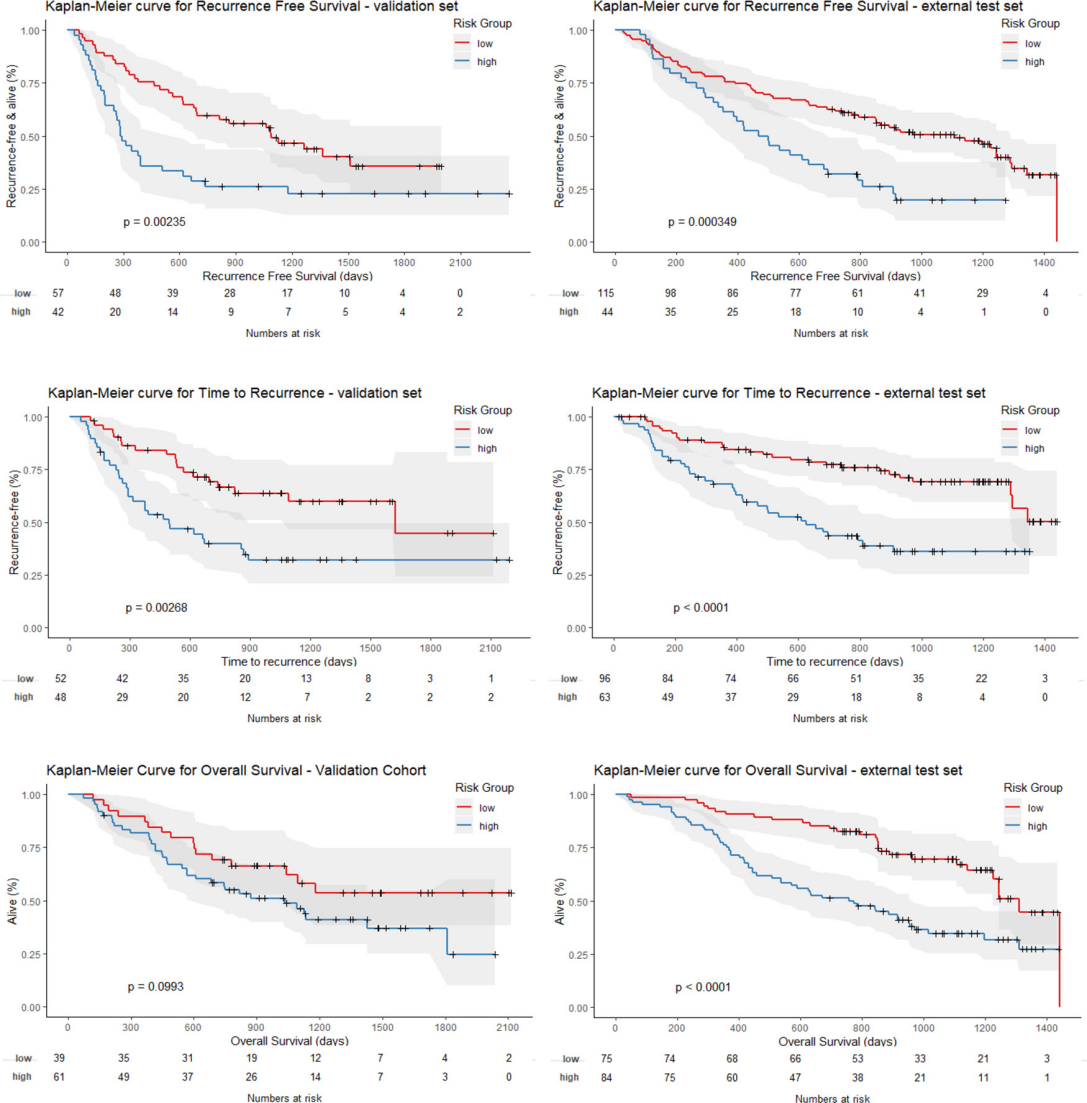
Kaplan Meier survival curves for low and high-risk groups in both validation and external test sets for each prediction model. *P*-values correspond to log-rank tests.

Kaplan Meier survival curves for validation and external test sets for each outcome are shown in Figure 3. Log-rank tests for the difference between survival curves was significant across the validation sets for RFS and recurrence (*P* < 0·01) and approached significance for OS (*P* = 0·0993). Stronger significance was demonstrated for all 3 outcomes in the external test set (*P* < 0·001), confirming a statistically significant difference in outcome between the high and low risk groups for prediction of each outcome. Classification metrics including balanced accuracy, F1 score, sensitivity, specificity, positive and negative predictive value along with Brier scores and calibration curves are detailed in Supplementary Tables 3 and 7 and Figure 1.

### Feature importance (subsection of results)

Supplementary Tables 4a-c show features ranked in order of importance for the KNN model for predicting RFS and for the component algorithms of the final ensemble models for predicting recurrence and OS. For RFS the top 5 features were PTV, size of the primary, the total number of fractions, whether treatment was with SBRT and the BED.

For predicting recurrence, feature importance for KNN and NB were identical, owing to the fact that these models do not have their own corresponding varImp package methods and instead use a filter approach where AUC is used to measure variable importance. For these models the top 5 features were also PTV, primary tumour size, number of fractions, SBRT treatment and BED. The RF model also ranked PTV and size of the primary, as well as the SUV of the primary, patient age and BMI in the top 5 most important features.

For predicting OS, the 8 features remaining after Kendall's rank correlation were PTV, size of the primary, BED, T1 stage, treatment with SBRT, treatment with chemoradiotherapy, whether there was nodal avidity and smoking status. There was a wide distribution of feature importance across features for the XGB and NNET models.

## Discussion

In this multicentre UK study of over 700 patients treated with curative-intent radiotherapy for NSCLC, we have compared machine learning algorithms and feature selection methods using routinely available clinical data and have developed and externally tested prediction models which are able to categorise patients into low and high risk for recurrence, RFS and OS, two years from the start of treatment. Such models may have future utility in personalised surveillance stratification, whereby only those at greatest risk have the most intensive follow-up.

AUCs for all three models are consistent between validation and external test sets though the recurrence model may demonstrate underfitting and the OS model overfitting in validation sets. Performance of the models is reasonable, demonstrating minor improvement on TNM-based prognostication, but highlight the need for more effective risk-stratification measures following radical radiotherapy for NSCLC – a call that may be better addressed with radiomic or deep-learning based image analysis.

For predicting RFS and recurrence, averaging across the machine learning algorithms, the best performing feature sets were those with no prior feature reduction. Unlike with -omics data where feature sets tend to be very large and are likely to contain noise and highly correlated features, this clinical feature set is comparatively small (*n* = 34) with all features possibly contributing to model performance to some degree, rendering feature reduction unnecessary. This is particularly the case where the models have inherent ability to handle multi-dimensional data and collinearity,^14^ for example LASSO and tree-based methods which have their own internal regularisation (L1-norm and number of estimators respectively). This was not the case for predicting OS however where Kendall's rank yielded the best performing feature set. One explanation for this is that OS had a lower event rate compared to recurrence and RFS, and therefore a higher risk of over-fitting to the majority class. As a result, feature reduction may play a more significant role in mitigating against this. Kendall's rank may have emerged as the best performing correlation method because it does not assume a normal distribution, is more robust to outliers and better suited to smaller datasets

Looking at the top 5 features ranked by importance for the final models or their component algorithms, these are features related to: size (PTV and size of the primary), stage (T1 stage and whether there was nodal avidity), treatment (BED, total number of fractions and treatment with SBRT) and also smoking status (ranked top 5 by the XGB and NNET algorithms contributing to the OS model) and SUV of the primary, BMI and age (ranked top 5 by the RF algorithm contributing to recurrence).

Whilst tumour size and stage are known prognostic factors for NSCLC,^12^^,^^15^^,^^16^ despite the high feature importance ranking in our models, strong evidence for tumour volume is lacking.^17^, ^18^, ^19^ The high importance of features related to treatment presumably reflects that our study included patients treated with SBRT as well as conventional radiotherapy and thus likely two cohorts of patients – those with early-stage disease that were older and not fit for surgery (treated with SBRT) and younger patients with more advanced disease (treated with conventional (chemo)radiotherapy). The former is less likely to experience recurrence both due to having early-stage disease but also as they are more likely to die of other comorbidities prior to recurrence. Smoking is linked to increased comorbidities and smoking status is known to be an independent prognostic factor for survival in NSCLC.^11^ A number of studies have demonstrated prognostic utility of PET SUV in NSCLC.^20^, ^21^, ^22^, ^23^ BMI and age are also likely to reflect the older and frailer cohort of patients described above that were likely to have been treated with SBRT.

A recent study also explored recurrence and OS prediction following curative-intent radiotherapy for NSCLC, using cox-proportional hazards models. Backwards stepwise elimination was used to select significant features resulting in 6 features for predicting recurrence and 5 features for predicting OS. External testing was not performed in this study, however on comparison of validation set AUCs with our external test set, our models demonstrate superior performance for predicting OS and similar for recurrence (0.717 vs 0.607 and 0.722 vs 0.72, respectively). Whilst this study did not rank feature importance, PS and nodal sampling with EBUS were common to both models^3^ and are known to hold prognostic value.^11^^,^^24^ Interestingly these were not considered in the top important features by our models.

Previous studies using clinical (non-imaging) data for outcome prediction following curative intent radiotherapy for NSCLC have generally focused on homogenous cohorts and OS as the key end-point, with few studies looking at recurrence or RFS.^5^, ^6^, ^7^, ^8^ This is likely due to accurate data on death being more readily accessible from national-level registries than recurrence data, however recurrence and RFS may offer more clinical utility than OS in stratifying follow-up, allowing clinicians to potentially intervene earlier to provide further, potentially curable treatment.

Earlier studies have focused on traditional statistical and machine learning models such as Cox-proportional hazards and SVMs.^3^^,^^5^, ^6^, ^7^, ^8^ Cox models have limitations in that the proportional hazard assumption and linearity of each variable must be satisfied, which can be difficult to do so with real-world data and may result in an inappropriate model fit. Secondly, in the context of tied samples, approximations are often employed to improve computational efficiency however these may result in significantly different results.^25^ Furthermore, survival analysis can experience multiple-testing problems in the context of high-dimensional data and may yield a high number of false positive significant features, which reduces reliability of analysis.^26^ Feature reduction based approaches coupled with more advanced machine learning algorithms may mitigate this issue and have been shown to provide more accurate alternatives to traditional Cox proportional-hazards models.^14^

Another potential benefit of exploring more advanced machine learning approaches is the ability of models to highlight “important” features as described above. Until recently prediction models tended to rely on clinician-determined input features thought to be of significance based on previous research. This approach is at risk of biasing and limiting choice of input features through human assumption. This can be mitigated to some degree by selecting a large number of potential input features and allowing machine learning models to select those that are best performing.^25^ These may not necessarily correlate with those thought to be most important by clinicians and may highlight features that were previously not considered.

A previous study compared discriminative performance of 6 classifiers in predicting a range of outcomes following radiotherapy for different tumours.^27^ Random forest and elastic net models showed the best overall discrimination, but no single classifier performed well across all datasets. The authors concluded that future investigators should benchmark models against random forest and elastic net models but that overall, informed preselection of a classifier based on specific datasets is advised. We build on this work by exploring a larger number of classifiers (including random forest and elastic net) together with feature reduction and believe that our methodology of applying this to our specific dataset is a robust approach to selecting the most suitable model.

Other strengths of our work are the multicentre design including a large cohort of over 700 patients from expert UK radiotherapy centres which is thus likely representative of UK (and probably European practice). Models were built using readily available clinical data with no requirement for access to, and complex pre-processing of imaging data. A broad set of features was used encompassing patient demographics, fitness, tumour characteristics and treatment parameters. The results are consistent across both validation and external test sets. Furthermore, this study included stage I-III disease treated with SBRT and conventional (chemo)radiotherapy, thus increasing clinical utility.

Whilst previous studies have demonstrated use of clinical data in developing prognostic prediction models following radiotherapy for NSCLC, none of these have compared multiple feature reduction methods and machine learning classification algorithms, as conveyed in our work. Finally, whilst these studies focus on survival analysis using few features, in this work, we have utilised the entire available feature set in our predictive modelling approach. Jochems et al. used a Bayesian network to predict 2-year survival using T-stage, N-stage, age and total tumour dose, with an external test set AUC of 0.66.^28^ With our wider range of features, our survival model achieves superior performance of 0.717.

Imaging and biological markers have been used for prognostication in NSCLC, however this requires complex pre-processing of imaging data or biological samples and may necessitate invasive procedures. The focus of our study was to use routinely available electronic health record clinical data to overcome these challenges. We believe that this methodology may be replicated across health systems, including those in resource-poor settings, using local clinical datasets to benefit surveillance stratification for patients following curative-intent radiotherapy globally. In addition, to demonstrate that there is a true benefit in using models built using imaging and biological markers, these should be compared with state-of-the-art clinical feature-based models. Our methodology has the potential to act as a clinical baseline for such models, as it provides reasonable predictions with clinically interpretable features. Furthermore, it may be combined with imaging and biological markers to enhance future models.

Weaknesses of our study include its retrospective nature, and reliance on data retrieved from the EPR and TPS of participating centres. As some participating sites were tertiary referral centres, some clinical information was not available if omitted at the time of initial referral. Owing to missing data, we had to omit 4 features: pre and post-treatment lymphocyte and neutrophil counts, which are known to have prognostic significance in this cohort.^29^, ^30^, ^31^ On the contrary, this was necessary as to maintain generalisability and utility, our models need to be built using features that are consistently available at other centres.

Treatment heterogeneity is a limitation that may be addressed by developing separate models for each treatment type. It would have been difficult to obtain sufficiently large datasets for a single treatment modality however, as we have curated novel datasets for this work. Additionally, within a single treatment modality there remains variation in dose and fractionation. Treatment modality, dose and number of fractions were features used for modelling to account for this and we therefore believe that our models allow for wider clinical utility. This work, including the novel datasets we have curated, may serve as a foundation for future models which are more specific to radiotherapy dose-fractionation schedules and treatment modalities.

Patients assumed to have NSCLC, without confirmed pathological diagnosis were included in this study as this is reflective of clinical practice, particularly for early-stage disease treated with SBRT. There is therefore potential inclusion of patients with benign disease or small cell lung cancer which may confound recurrence and survival rates, however our models were provided with this information and such patients would still benefit from post-treatment risk-stratification. Our study did not include patients treated with tri-modality therapy (surgery before or after radiotherapy) and we do not have data on patients treated with adjuvant Durvalumab as per the PACIFIC trial^32^ or for oligometastatic recurrence which may impact on recurrence or survival.

In developing our ensemble models, we used averaged predictions of the component algorithms, however more complex ensemble architectures such as random forest or majority weighting may yield superior results. Finally, owing to the patient cohort used for training and validation, whilst our models are reproducible, they are unlikely to be generalisable outside the UK or Europe. Replication of these data in future international prospective clinical trials are warranted.

In this study we have compared multiple machine learning algorithms and feature selection methods to develop prognostic prediction models for recurrence, RFS and OS following curative-intent radiotherapy for NSCLC. Our models demonstrate reasonable performance exceeding traditional methods and show consistency across validation and external test sets. More advanced imaging-based models may surpass this approach but there is potential value in use of routinely available clinical data. This robust and ready to use machine learning method, validated and externally tested, sets the stage for future clinical trials entailing quantitative personalised risk-stratification and surveillance following curative-intent radiotherapy for NSCLC.

## Contributors

Sumeet Hindocha, Kristofer Linton-Reid, Merina Ahmed, Richard Lee, and Eric Aboagye were involved in the study design.

Sumeet Hindocha, Thomas Charlton, Benjamin Hunter, and Charleen Chan collected the data.

Sumeet Hindocha analysed and interpreted the data.

Sumeet Hindocha undertook literature review and wrote the initial draft.

Sumeet Hindocha and Kristofer Linton-Reid undertook radiomic analysis.

Kristofer Linton-Reid, Emily Robinson, Matthew Orton, and Matthew Blackledge provided statistical and machine learning advice.

Merina Ahmed, Shahreen Ahmad, Fiona McDonald, Imogen Locke, Danielle Power and Richard Lee provided clinical advice.

All authors reviewed, contributed to, and approved the manuscript. All authors had access to the data and Sumeet Hindocha and Richard Lee directly accessed and verified the underlying data reported in the manuscript. Sumeet Hindocha, Richard Lee and Eric Aboagye were responsible for the decision to submit the manuscript.

## Declaration of interests

Richard Lee receives funding from Cancer Research UK, Innovate UK (co-funded with Roche and Optellum), NIHR and RM Partners outside of this work. He is Joint Clinical Lead for the NHS England Lung Health Checks programme and a National Specialty Lead for the NIHR, and receives funding directly to his institution, outside of this work for these roles. He receives consulting fees from the Royal Marsden Private Care, and honoraria from Cancer Research UK as a member of the Early Diagnosis grants peer review panel, not related to this work.

## Appendix Supplementary materials

Image, application 1

Image, application 2
