# Supplementary material for: A comparison of machine learning methods for predicting recurrence and death after curative-intent radiotherapy for non-small cell lung cancer: Development and validation of multivariable clinical prediction models
Source: eBioMedicine. 2022 Mar 3;77:103911. doi: 10.1016/j.ebiom.2022.103911 (PMC8897583; doi:10.1016/j.ebiom.2022.103911)
Supplement: Supplementary file 1 [file mmc1.docx]

The caption for the supplementary file is:

“Supplementary material including supplementary tables and figures and TRIPOD checklist”.
