# Supplementary material for: A comparison of machine learning methods for predicting recurrence and death after curative-intent radiotherapy for non-small cell lung cancer: Development and validation of multivariable clinical prediction models
Source: eBioMedicine. 2022 Mar 3;77:103911. doi: 10.1016/j.ebiom.2022.103911 (PMC8897583; doi:10.1016/j.ebiom.2022.103911)
Supplement: Supplementary file 2 [file mmc2.docx]

**Supplementary Material:**

**Table of Contents:**

Summary of machine learning algorithms

Summary of feature selection methods

Supplementary Table 1 – Hyper-parameters

Supplementary Table 2 – Feature sets

Supplementary Table 3 – Classification metrics

Supplementary Table 4 – Feature Importance for RFS

Supplementary Table 5: Features Importance for recurrence

Supplementary Table 6: Features Importance for OS

Supplementary Table 7: Brier scores for the validation and external test set for each prediction model

Supplementary Figure 1: Calibration curves

Tripod Checklist

**Summary of machine learning algorithms:**

The models used in this study are supervised classification algorithms. A summary of each is provided below.

GLM – generalised linear model is a generalisation of linear regression to modelling dependencies between predictors and dependent features. Logistic regression is a form of GLM used in this study. It uses the logistic sigmoid function to return a probability value which can then be mapped to two or more separate classes^1^.

SVM (linear and radial) – support vector machines plot training samples and assigns a hyperplane (decision boundary) to separate these into classes. The optimal hyperplane is that which maximises the distance between data-points. Where data is non-linear it is transformed to a higher dimensional feature space and a non-linear (e.g. radial) decision boundary is then applied to separate classes^2^.

KNN – K-nearest neighbours assumes that data points that are close to each other are of the same class. It takes a defined number (k) of training samples closest in Euclidian distance to a new point and predicts a class based on these^3^.

RF & XGB – Random Forest and Extreme Gradient Boosting Machines are ensemble decision-tree based models. RF uses bagging and feature variability when building each decision tree to create an uncorrelated forest whose overall prediction is more accurate than each individual tree^4^. XGB by contrast takes a boosting approach whereby trees are grown iteratively using information from a previously grown tree, to minimise the error of previous trees^5^.

MDA – a discriminant analysis method where each class is assumed to be a gaussian mixture of subclasses^6^.

PLS – Partial Least Squares is a multivariate linear regression model which forms linear combinations of features in a supervised manner. It is able to handle datasets with large numbers of features, high collinearity between features and small numbers of observations^7^.

NNET – the “nnet” package fits a single-layer feed-forward neural network^8^.

NB – the Naïve-Bayes model is based on Bayes-theorem and assumes no interdependence between variables^9^.

**Summary of feature reduction methods:**

LASSO (Least Absolute Shrinkage and Selection Operator) and Elastic Net regression are examples of regularisation methods^10^. Here a penalty is applied to the coefficient which multiplies each feature in a linear model and results in less overfitting and improved generalisation. LASSO uses the L1 regularisation penalty to force some coefficients to zero. This eliminates some features leaving a subset of predictors that are thought to be important. Alpha = 1. Elastic Net incorporates penalties from both L1 and L2 (ridge regression) regularisation. Here, Alpha =0·5. The glmnet package was used to perform both LASSO and Elastic-net regression.

Pearson, Spearman’s rank and Kendall’s rank correlation are “filter” feature selection methods which rely only on the characteristics of feature independently of any machine learning model. Pearson’s correlation assumes data is parametric and linear. Spearman and Kendall’s rank are non-parametric and assume a monotonic relationship between variables. Kendall’s rank is preferred to Spearman’s where dataset have a limited number of observations or contain outliers. For our study we used the corr package and specified that the top 25% (8) features be included in the feature sets following Pearson’s, Spearman’s and Kendall’s rank correlation.

Univariate LR – apply univariate logistic regression for each feature to the outcome variable and select only those features with a certain p-value. We used the glm package with a p-value <0·005, adjusted for multiple comparisons with the Benjamini & Hochberg method^1^.

RFE – Recursive Feature Elimination is a “wrapper” feature selection method which fits a model and removes the weakest feature until a specified number of features is reached. Cross-validation is used to score different feature subsets and select the best scoring collection of features to identify the optimal number of features. Features are ranked by the model’s feature importance. By recursively eliminating features iteratively the collinearity is reduced^11^. We used the rfeControl package with a random forest model and 10-fold cross validation with 5 repeats.

**Supplementary Table 1:** Hyperparameters used for the final KNN model (predicting RFS) or component algorithms for the ensemble models (predicting recurrence and OS).

| Model | Library | Hyperparameter |
| --- | --- | --- |
| RFS | | |
| - KNN | *knn* | K = 43 |
| Recurrence | | |
| - KNN | *knn* | K = 37 |
| - NB | *naïve_bayes* | laplace = 0, usekernel = FALSE, adjust = 1 |
| - RF | *rf* | mtry = 2 |
| OS | | |
| - XGB | *xgbTree* | nrounds = 1000, max_depth = 6, eta = 0·001, gamma = 0, colsample_bytree = 0·5, min_child_weight = 1, subsample = 0·6 |
| - NNET | *nnet* | size = 1, decay = 0·06812921 |
| - MDA | *mda* | Subclasses = 3 |

**Supplementary Table 2:** The full feature set after data pre-processing contained 12 continuous and 22 binary features. Continuous features are indicated with “*”. Features that remained after each feature reduction method for each of the measured outcomes are indicated with an X.

| **Full Feature Set** | **Recurrence Free Survival** | | | | | | | **Recurrence** | | | | | | | **Overall Survival** | | | | | | |
| --- | --- | --- | --- | --- | --- | --- | --- | --- | --- | --- | --- | --- | --- | --- | --- | --- | --- | --- | --- | --- | --- |
|  | **LASSO** | **E-Net** | **RFE** | **Pearson** | **Spearman** | **Kendall** | **Univariate LR** | **LASSO** | **E-Net** | **RFE** | **Pearson** | **Spearman** | **Kendall** | **Univariate LR** | **LASSO** | **E-Net** | **RFE** | **Pearson** | **Spearman** | **Kendall** | **Univariate LR** |
| **age*** |  |  |  |  |  |  |  | X | X |  |  |  |  | X |  |  |  |  |  |  |  |
| **ptv*** | X | X | X | X | X | X | X | X | X | X | X | X | X | X | X | X | X | X | X | X | X |
| **sizeprimary*** | X | X | X | X | X | X | X |  |  |  |  | X |  | X |  | X | X | X | X | X | X |
| **suvprimary*** |  |  | X |  |  |  |  |  |  |  |  |  | X |  |  |  | X |  |  |  |  |
| **suvnodal*** |  |  |  |  |  |  |  |  |  | X |  |  |  | X |  |  | X |  |  |  |  |
| **pstoffdays*** |  |  |  |  |  |  |  |  |  |  |  |  |  |  |  | X | X |  |  |  |  |
| **bmi*** |  |  |  |  |  |  |  |  |  |  |  |  |  |  |  |  | X |  |  |  |  |
| **fev1*** |  |  |  |  |  |  |  |  |  |  |  |  |  |  | X | X | X |  |  |  |  |
| **tlco*** |  |  |  |  |  |  |  |  |  |  |  |  |  |  |  |  | X |  |  |  |  |
| **totaldose*** |  |  |  |  |  |  |  |  |  |  | X | X | X | X |  |  | X | X | X |  |  |
| **totalfrac*** |  |  |  | X | X | X | X |  |  |  | X |  |  | X | X | X | X | X | X | X | X |
| **bed*** |  |  | X | X | X |  | X |  |  |  |  |  |  |  |  |  | X |  |  |  |  |
| **sex_M** | X | X |  |  |  |  |  |  |  |  |  |  |  |  | X | X | X |  |  |  |  |
| **perfstat_0** |  |  |  |  |  |  |  |  |  |  |  |  |  |  |  |  | X |  |  |  |  |
| **perfstat_1** |  |  |  |  |  |  |  |  |  |  |  |  |  |  |  |  |  |  |  |  |  |
| **perfstat_2** |  |  |  |  |  |  |  |  |  |  |  |  |  |  | X | X | X | X | X | X |  |
| **smoking_stat_Never** | X | X |  |  |  |  |  | X | X |  | X | X | X | X | X | X | X | X | X | X | X |
| **tstage_1** | X | X | X | X | X | X | X |  |  |  |  |  |  |  |  |  | X |  |  |  |  |
| **tstage_2** |  |  |  |  |  |  |  |  |  |  |  |  |  |  | X | X | X |  |  |  |  |
| **tstage_3** |  | X | X |  |  |  |  |  |  |  |  |  |  | X |  |  | X |  |  |  |  |
| **tstage_4** |  |  |  |  |  |  | X |  |  |  | X | X | X | X |  |  | X |  |  |  |  |
| **nstage_0** |  |  |  | X | X | X | X |  |  |  |  |  |  |  |  |  | X |  |  |  |  |
| **nstage_1** |  |  |  |  |  |  |  |  |  |  |  |  |  |  |  |  | X |  |  |  |  |
| **nstage_2** |  |  |  |  |  |  |  | X | X |  |  |  |  |  |  |  |  |  |  |  |  |
| **nstage_3** |  |  |  |  |  |  |  | X | X | X | X | X | X | X |  |  | X | X | X | X |  |
| **nodal_avidity_Yes** |  | X |  |  |  | X | X |  |  |  |  |  |  |  |  |  | X |  |  |  |  |
| **ebus_Yes** |  |  |  |  |  |  |  |  |  |  |  |  |  |  |  |  | X |  |  |  |  |
| **morphology_Adenocarcinoma** |  |  |  |  |  |  |  |  |  |  |  |  |  |  |  |  |  |  |  |  |  |
| **morphology_No pathological diagnosis** |  |  |  |  |  |  |  |  |  |  |  |  |  |  |  |  | X |  |  |  |  |
| **morphology_Other** |  |  |  |  |  |  |  |  |  |  |  |  |  |  |  | X | X |  |  |  |  |
| **morphology_Squamous** |  |  |  |  |  |  |  |  |  |  |  |  |  |  |  |  | X |  |  |  |  |
| **treatment_ChemoRT** |  |  |  | X | X | X | X |  |  |  | X | X | X | X |  |  | X |  |  | X |  |
| **treatment_Conventional RT** |  |  |  |  |  |  |  |  |  |  |  |  |  |  |  |  | X |  |  |  |  |
| **treatment_SBRT** | X | X | X | X | X | X | X | X | X |  | X | X | X | X |  |  | X | X | X | X | X |

**Supplementary Table 3:** Results of classification for the validation and external test sets. The classification threshold used was derived from the Youden Index of the validation set ROC curve for each model.

| RFS model | Validation Set | External Test set |
| --- | --- | --- |
| Youden Index | 0·616 |  |
| Accuracy | 0·647 | 0·635 |
| Accuracy 95% CI | 0·544 – 0·740 | 0·555 – 0·71 |
| No Information Rate | 0·535 | 0·535 |
| P-value (Acc > NIR) | 0·017 | 0·007 |
| Balanced Accuracy | 0·653 | 0·62 |
| F1 Score | 0·632 | 0·508 |
| Sensitivity / Recall | 0·566 | 0·405 |
| Specificity | 0·739 | 0·835 |
| PPV / Precision | 0·714 | 0·682 |
| NPV | 0·597 | 0·617 |
| Recurrence Ensemble model | | |
| Youden Index | 0·584 |  |
| Accuracy | 0·65 | 0·692 |
| Accuracy 95% CI | 0·548 – 0·743 | 0·614 – 0·763 |
| No Information Rate | 0·57 | 0·660 |
| P-value (Acc > NIR) | 0·064 | 0·227 |
| Balanced Accuracy | 0·65 | 0·677 |
| F1 Score | 0·615 | 0·581 |
| Sensitivity / Recall | 0·651 | 0·630 |
| Specificity | 0·649 | 0·724 |
| PPV / Precision | 0·583 | 0·540 |
| NPV | 0·712 | 0·792 |
| OS Ensemble model | | |
| Youden Index | 0·343 |  |
| Accuracy | 0·67 | 0·648 |
| Accuracy 95% CI | 0·569 – 0·761 | 0·568 – 0·722 |
| No Information Rate | 0·63 | 0·660 |
| P-value (Acc > NIR) | 0·236 | 0·665 |
| Balanced Accuracy | 0·71 | 0·675 |
| F1 Score | 0·66 | 0.594 |
| Sensitivity / Recall | 0·865 | 0·759 |
| Specificity | 0·556 | 0·591 |
| PPV / Precision | 0·533 | 0·488 |
| NPV | 0·875 | 0·827 |

**Supplementary Table 4:** Features ranked in order of importance for the KNN model for predicting RFS.

| Predicting RFS – KNN with no prior feature reduction | | |
| --- | --- | --- |
| Feature | | **Importance** |
| 1 | ptv | 0·702 |
| 2 | sizeprimary | 0·667 |
| 3 | totalfrac | 0·637 |
| 4 | treatment_SBRT | 0·635 |
| 5 | bed | 0·630 |
| 6 | suvprimary | 0·622 |
| 7 | tstage_1 | 0·617 |
| 8 | suvnodal | 0·609 |
| 9 | nstage_0 | 0·608 |
| 10 | nodalavidity_Yes | 0·604 |
| 11 | treatment_ChemoRT | 0·604 |
| 12 | tstage_4 | 0·571 |
| 13 | totaldose | 0·565 |
| 14 | sex_M | 0·560 |
| 15 | nstage_2 | 0·558 |
| 16 | tstage_3 | 0·552 |
| 17 | morphology_No pathological diagnosis | 0·551 |
| 18 | pstoffdays | 0·549 |
| 19 | bmi | 0·545 |
| 20 | age | 0·541 |

**Supplementary Table 5:** Features ranked in order of importance for the component algorithms of the final ensemble model for predicting recurrence.

| Predicting Recurrence – components of final ensemble model, no prior feature reduction | | | | | |
| --- | --- | --- | --- | --- | --- |
| KNN & NB | | | **RF** | | |
| Feature | | **Importance** | **Feature** | | **Importance** |
| 1 | ptv | 0·702 | 1 | ptv | 13·115 |
| 2 | totalfrac | 0·646 | 2 | suvprimary | 10·529 |
| 3 | treatment_SBRT | 0·640 | 3 | sizeprimary | 10·037 |
| 4 | sizeprimary | 0·636 | 4 | bmi | 9·669 |
| 5 | bed | 0·630 | 5 | age | 9·468 |
| 6 | nodalavidity_Yes | 0·625 | 6 | tlco | 9·408 |
| 7 | suvnodal | 0·622 | 7 | fev1 | 9·342 |
| 8 | tstage_1 | 0·621 | 8 | pstoffdays | 8·132 |
| 9 | nstage_0 | 0·620 | 9 | suvnodal | 7·070 |
| 10 | treatment_ChemoRT | 0·618 | 10 | bed | 5·077 |
| 11 | suvprimary | 0·604 | 11 | totalfrac | 4·909 |
| 12 | age | 0·590 | 12 | totaldose | 2·865 |
| 13 | totaldose | 0·576 | 13 | tstage_1 | 2·726 |
| 14 | tstage_4 | 0·566 | 14 | treatment_ChemoRT | 2·533 |
| 15 | nstage_2 | 0·563 | 15 | sex_M | 2·405 |
| 16 | tlco | 0·561 | 16 | nodalavidity_Yes | 2·389 |
| 17 | morphology_No pathological diagnosis | 0·551 | 17 | treatment_SBRT | 2·365 |
| 18 | ebus_Yes | 0·550 | 18 | ebus_Yes | 2·230 |
| 19 | fev1 | 0·547 | 19 | morphology_Adenocarcinoma | 2·141 |
| 20 | nstage_3 | 0·542 | 20 | nstage_0 | 2·119 |

**Supplementary Table 6:** Features ranked in order of importance for the component algorithms of the final ensemble model for predicting OS.

| Predicting OS – components of final ensemble model, after Kendall Rank Feature Reduction | | | | | | | | |
| --- | --- | --- | --- | --- | --- | --- | --- | --- |
| MDA | | | **XGB** | | | **NNET** | | |
| Feature | | **Importance** | **Feature** | | **Importance** | **Feature** | | **Importance** |
| 1 | ptv | 0·662 | 1 | ptv | 0·483 | 1 | smokingstat_Never | 44·259 |
| 2 | sizeprimary | 0·630 | 2 | sizeprimary | 0·302 | 2 | treatment_SBRT | 19·133 |
| 3 | bed | 0·613 | 3 | bed | 0·085 | 3 | ptv | 15·269 |
| 4 | tstage_1 | 0·603 | 4 | smokingstat_Never | 0·047 | 4 | bed | 10·607 |
| 5 | treatment_SBRT | 0·597 | 5 | nodalavidity_Yes | 0·031 | 5 | tstage_1 | 6·005 |
| 6 | nodalavidity_Yes | 0·578 | 6 | tstage_1 | 0·026 | 6 | treatment_ChemoRT | 2·256 |
| 7 | treatment_ChemoRT | 0·575 | 7 | treatment_SBRT | 0·013 | 7 | sizeprimary | 1·430 |
| 8 | smokingstat_Never | 0·548 | 8 | treatment_ChemoRT | 0·013 | 8 | nodalavidity_Yes | 1·042 |

**Supplementary Table 7:** Brier scores for the validation and external test set for each prediction model.

| Model | Validation Set | External Test set |
| --- | --- | --- |
| RFS | 0·32 | 0·32 |
| Recurrence | 0·24 | 0·2 |
| OS | 0·2 | 0·2 |

**Supplementary Figure 1.** Calibration curves for validation and external test sets for each prediction model. The data is divided into bins, with the y-axis representing the distribution of positive cases in each bin while the x-axis the probability as predicted by the classifier. The closer the resulting calibration curve is to the reference line. the better the model's predictions reflect the actual class distribution in the dataset.


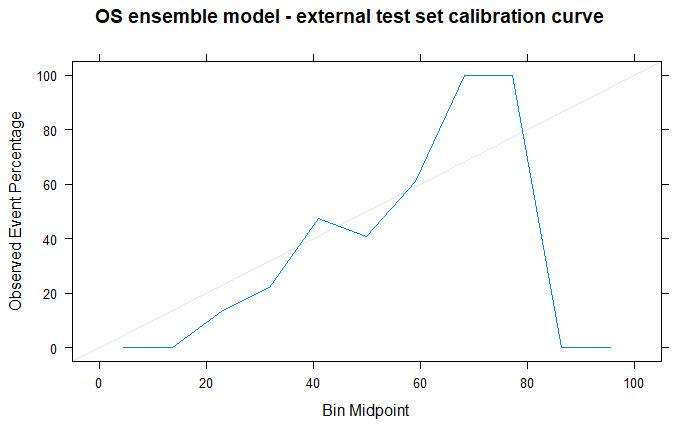

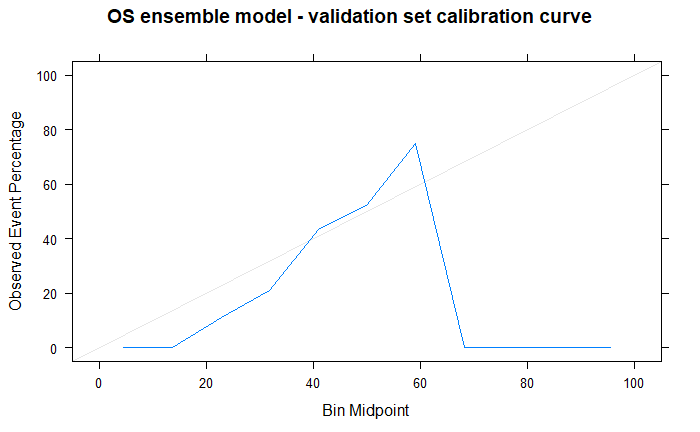

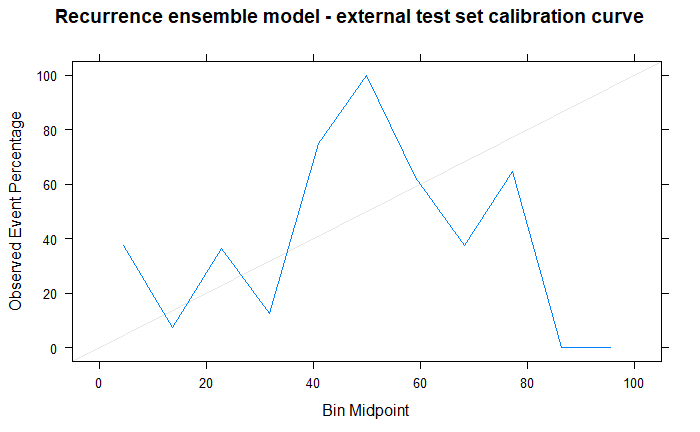

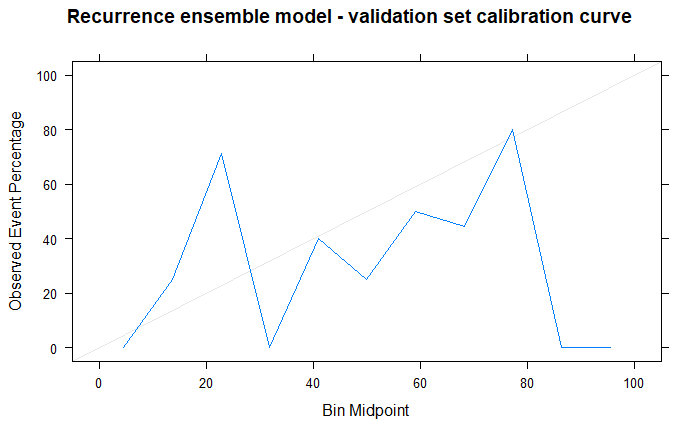

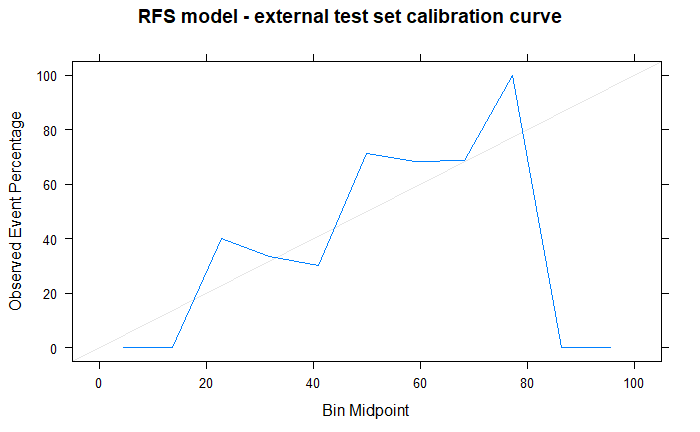

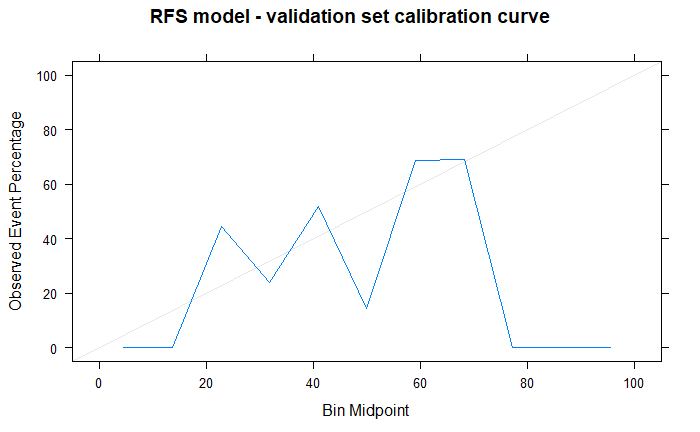


**Tripod Checklist:**

| **Section/Topic** | **Item** |  | **Checklist Item** | **Page** |
| --- | --- | --- | --- | --- |
| **Title and abstract** | | | | |
| Title | 1 | D;V | Identify the study as developing and/or validating a multivariable prediction model, the target population, and the outcome to be predicted. | X |
| Abstract | 2 | D;V | Provide a summary of objectives, study design, setting, participants, sample size, predictors, outcome, statistical analysis, results, and conclusions. | X |
| **Introduction** | | | | |
| Background and objectives | 3a | D;V | Explain the medical context (including whether diagnostic or prognostic) and rationale for developing or validating the multivariable prediction model, including references to existing models. | X |
|  | 3b | D;V | Specify the objectives, including whether the study describes the development or validation of the model or both. | X |
| **Methods** | | | | |
| Source of data | 4a | D;V | Describe the study design or source of data (e.g., randomized trial, cohort, or registry data), separately for the development and validation data sets, if applicable. | X |
|  | 4b | D;V | Specify the key study dates, including start of accrual; end of accrual; and, if applicable, end of follow-up. | X |
| Participants | 5a | D;V | Specify key elements of the study setting (e.g., primary care, secondary care, general population) including number and location of centres. | X |
|  | 5b | D;V | Describe eligibility criteria for participants. | X |
|  | 5c | D;V | Give details of treatments received, if relevant. | X |
| Outcome | 6a | D;V | Clearly define the outcome that is predicted by the prediction model, including how and when assessed. | X |
|  | 6b | D;V | Report any actions to blind assessment of the outcome to be predicted. |  |
| Predictors | 7a | D;V | Clearly define all predictors used in developing or validating the multivariable prediction model, including how and when they were measured. | X |
|  | 7b | D;V | Report any actions to blind assessment of predictors for the outcome and other predictors. |  |
| Sample size | 8 | D;V | Explain how the study size was arrived at. |  |
| Missing data | 9 | D;V | Describe how missing data were handled (e.g., complete-case analysis, single imputation, multiple imputation) with details of any imputation method. | X |
| Statistical analysis methods | 10a | D | Describe how predictors were handled in the analyses. | X |
|  | 10b | D | Specify type of model, all model-building procedures (including any predictor selection), and method for internal validation. | X |
|  | 10c | V | For validation, describe how the predictions were calculated. | X |
|  | 10d | D;V | Specify all measures used to assess model performance and, if relevant, to compare multiple models. | X |
|  | 10e | V | Describe any model updating (e.g., recalibration) arising from the validation, if done. |  |
| Risk groups | 11 | D;V | Provide details on how risk groups were created, if done. | X |
| Development vs. validation | 12 | V | For validation, identify any differences from the development data in setting, eligibility criteria, outcome, and predictors. | X |
| **Results** | | | | |
| Participants | 13a | D;V | Describe the flow of participants through the study, including the number of participants with and without the outcome and, if applicable, a summary of the follow-up time. A diagram may be helpful. | X |
|  | 13b | D;V | Describe the characteristics of the participants (basic demographics, clinical features, available predictors), including the number of participants with missing data for predictors and outcome. | X |
|  | 13c | V | For validation, show a comparison with the development data of the distribution of important variables (demographics, predictors and outcome). | X |
| Model development | 14a | D | Specify the number of participants and outcome events in each analysis. | X |
|  | 14b | D | If done, report the unadjusted association between each candidate predictor and outcome. |  |
| Model specification | 15a | D | Present the full prediction model to allow predictions for individuals (i.e., all regression coefficients, and model intercept or baseline survival at a given time point). |  |
|  | 15b | D | Explain how to the use the prediction model. |  |
| Model performance | 16 | D;V | Report performance measures (with CIs) for the prediction model. | X |
| Model-updating | 17 | V | If done, report the results from any model updating (i.e., model specification, model performance). |  |
| **Discussion** | | | | |
| Limitations | 18 | D;V | Discuss any limitations of the study (such as nonrepresentative sample, few events per predictor, missing data). | X |
| Interpretation | 19a | V | For validation, discuss the results with reference to performance in the development data, and any other validation data. | X |
|  | 19b | D;V | Give an overall interpretation of the results, considering objectives, limitations, results from similar studies, and other relevant evidence. | X |
| Implications | 20 | D;V | Discuss the potential clinical use of the model and implications for future research. | X |
| **Other information** | | | | |
| Supplementary information | 21 | D;V | Provide information about the availability of supplementary resources, such as study protocol, Web calculator, and data sets. | X |
| Funding | 22 | D;V | Give the source of funding and the role of the funders for the present study. | X |

*Items relevant only to the development of a prediction model are denoted by D, items relating solely to a validation of a prediction model are denoted by V, and items relating to both are denoted D;V. We recommend using the TRIPOD Checklist in conjunction with the TRIPOD Explanation and Elaboration document.

**Supplementary Material References:**

1 Venables WN, Ripley BD. Generalized Linear Models. In: Modern Applied Statistics with S. New York, NY: Springer New York, 2002: 183–210.

2 Karatzoglou A, Hornik K, Smola A, Zeileis A. kernlab - An S4 package for kernel methods in R. *J Stat Softw* 2004; **11**: 1–20.

3 Venables WN, Ripley BD. Classification. In: Modern Applied Statistics with S. New York, NY: Springer New York, 2002: 331–51.

4 Breiman L. Random forests. *Mach Learn* 2001; **45**: 5–32.

5 Chen T, Guestrin C. XGBoost: A scalable tree boosting system. In: Proceedings of the ACM SIGKDD International Conference on Knowledge Discovery and Data Mining. New York, NY, USA: ACM, 2016: 785–94.

6 Leisch F, Hornik K, Ripley Balasubrama BD. Package ‘mda’. 2020. https://cran.r-project.org/web/packages/mda/mda.pdf (accessed Aug 13, 2021).

7 Mevik B-H, Wehrens R. Introduction to the pls Package. Help Sect. ‘pls’ Packag. RStudio Softw. 2015; : 1–23.

8 Riplley B, Venables B, Bates DM, Firth D, Hornik K, Gebhardt A. Package ‘MASS’. Support Functions and Datasets for Venables and Ripley’s MASS. Doc. Free. available internet http//www. r-project. org. 2018; : 169.

9 Michal Majka. High performance implementation of the Naive Bayes algorithm. R package naivebayes. Version 0.9.7. 2019. https://majkamichal.github.io/naivebayes/ (accessed Aug 13, 2021).

10 Friedman J, Hastie T, Tibshirani R. Regularization paths for generalized linear models via coordinate descent. *J Stat Softw* 2010; **33**: 1–22.

11 Guyon I, Weston J, Barnhill S, Vapnik V. Gene selection for cancer classification using support vector machines. *Mach Learn* 2002; **46**: 389–422.
